# Phylogeography and systematics of the westernmost Italian *Dolichopoda* species (Orthoptera, Rhaphidophoridae)

**DOI:** 10.3897/zookeys.437.7917

**Published:** 2014-08-28

**Authors:** Giuliana Allegrucci, Mauro Rampini, Claudio Di Russo, Enrico Lana, Sara Cocchi, Valerio Sbordoni

**Affiliations:** 1 Dipartimento di Biologia, Università di Roma Tor Vergata, via della ricerca scientifica s.n.c. 00133 Rome, Italy; 2 Dipartimento di Biologia e Biotecnologie “Charles Darwin”, Università di Roma La Sapienza, Viale dell’Università 32, 00100 Rome, Italy; 3 Gruppo Speleologico Piemontese (Torino) and Gruppo Speleologico Alpi Marittime (Cuneo)

**Keywords:** Westernmost *Dolichopoda* species, barcoding, systematics

## Abstract

The genus *Dolichopoda* (Orthoptera; Rhaphidopohoridae) is present in Italy with 9 species distributed from northwestern Italy (Piedmont and Liguria) to the southernmost Apennines (Calabria), occurring also in the Tyrrhenian coastal areas and in Sardinia. Three morphologically very close taxa have been described in Piedmont and Liguria, i.e., *D. ligustica ligustica*, *D. ligustica septentrionalis* and *D. azami azami*.

To investigate the delimitation of the northwestern species of *Dolichopoda*, we performed both morphological and molecular analyses. Morphological analysis was carried out by considering diagnostic characters generally used to distinguish different taxa, as the shape of epiphallus in males and the subgenital fig in females. Molecular analysis was performed by sequencing three mitochondrial genes, 12S rRNA, 16S rRNA, partially sequenced and the entire gene of COI.

Results from both morphological and molecular analyses highlighted a very homogeneous group of populations, although genetically structured. Three haplogroups geographically distributed could be distinguished and based on these results we suggest a new taxonomic arrangement. All populations, due to the priority of description, should be assigned to *D. azami azami* Saulcy, 1893 and to preserve the names *ligustica* and *septentrionalis*, corresponding to different genetic haplogroups, we assign them to *D. azami ligustica* stat. n. Baccetti & Capra, 1959 and to *D. azami septentrionalis* stat. n. Baccetti & Capra, 1959.

## Introduction

*Dolichopoda* is a Mediterranean genus, consisting of more than fifty species distributed throughout the North Mediterranean regions from the Pyrenees to Transcaucasia and north Iran (Di Russo and Rampini 2014). The highest species diversity is present in insular and peninsular Greece where most of the species have been described (Di Russo et al. 2007). However, many species are present also in Italy. In particular, nine species of *Dolichopoda* occur in Italy, where they range from the Maritime Alps to the southern tip of the Italian peninsula, excluding the oriental Alps, the Liguria Apennines, the Apuane Alps and the southern Apulia. Most species of this genus are strictly dependent upon caves. However, especially in the northern part of the range, several populations inhabit cave-like habitat such as rock-crevices and ravines and individuals can be observed, during the night, outside in moist or mesic woods. In peninsular Italy, *Dolichopoda* populations often live in cellars, catacombs, aqueducts, Etruscan tombs and other similar man-made hypogean environments. Population sizes can be small and constant over long periods, at least in natural caves (Carchini et al. 1983; Sbordoni et al. 1987).

Based on morphology three subgenera have been described in Italy: *Dolichopoda (Dolichopoda)*, *Dolichopoda (Chopardina)* and *Dolichopoda (Capraiacris)* (Baccetti 1975; Baccetti and Capra 1959, 1970). One of the main morphological differences between these sub-genera is presence or absence of spinulation on the legs. The subgenus *Dolichopoda* includes the highest number of species distributed throughout the range of the genus, except for some coastal areas and it is characterized by the presence of spines on the anterior tibiae. Species belonging to *Chopardina* sub-genus are mostly found in insular and peninsular Tuscany and Sardinia in Italy and in Corsica, with two other species found in Greece (Macedonia and Etolia Akarnania). *Chopardina* species are characterized by the presence of several spines also on the hind femurs. The subgenus *Capraiacris* includes only two species, restricted to the Giglio Island and Monte Argentario in the Tuscan archipelago. Its two species (*Dolichopoda aegilion* and *Dolichopoda baccettii*) are distinct from all the other species because of the lack of spines on the anterior tibiae and on the hind femur. However, studies based on molecular characters did not support the subdivision in sub-genera, showing that the subgenus *Chopardina* is polyphyletic and the subgenus *Dolichopoda* is paraphyletic (Allegrucci et al. 2005). Since the main morphological character defining the subgenera is the presence/absence of spines on the appendages, Allegrucci et al. (2005) concluded that such character is an adaptive character subject to homoplasy. Based on these observations we decided to not consider the subdivision in sub-genera of the species belonging to genus *Dolichopoda*.

Based on molecular phylogenetic reconstruction (Allegrucci et al. 2005; 2009; 2011) the current distribution of *Dolichopoda* species has been essentially shaped by the palaeogeographic and climatic events occurred in the Mediterranean region, starting from Late Miocene. However, also adaptation to cave life seems to have played an important role in this process. In particular, the end of Messinian salinity crisis would mark the geographic separation of the epigean forest populations and the beginning of dispersal toward the west Mediterranean region. The migration and /or the isolation of the different insular populations might have also been favored by marine regressions and transgressions, during the Plio-Pleistocene. This era has been also characterized by climatic shifts during alternating glacial and interglacial periods. The sylvicolous ancestors of *Dolichopoda* might have used caves as refugia during the unfavorable climatic conditions, beginning their adaptation to subterranean habitat. Therefore, the current distribution of *Dolichopoda* can be explained by a combination of both vicariance and dispersal events, with many processes occurring in ancestral epigean populations before the invasion of the subterranean environment.

The most western Italian species are represented by *Dolichopoda ligustica*, and *Dolichopoda azami*. Two subspecies are attributed to *Dolichopoda ligustica*, i.e., *Dolichopoda ligustica ligustica* and *Dolichopoda ligustica septentrionalis*. The first one is widespread in the Southern Piedmont (Maritime Alps) and in the north-western Liguria while the range of *Dolichopoda ligustica septentrionalis* is northernmost and limited to a small area in Val di Lanzo valley, close to Turin, and in some easternmost caves, close to Bergamo. *Dolichopoda azami* Saulcy, 1893 is widespread in the south eastern France and observed also in Piedmont in a cave in Grana Valley (Baccetti and Capra 1959). The systematic position of *Dolichopoda azami* has long been discussed (Finot 1890; Azam 1893; Chopard 1922, 1951) until Baccetti (1966) reclassified the systematics of the western *Dolichopoda* species, based on the morphology of the epiphallus and of the tergum 10, the most important characters for the systematics of the genus *Dolichopoda*.

*Dolichopoda ligustica* and *Dolichopoda azami* have very similar morphologies with little variation in the characters on which the systematics are based. To better understand the differentiation between these two species and the limits of the ranges occupied by each of them, we carried out a study on several populations of *Dolichopoda ligustica* and *Dolichopoda azami*, both at morphological and molecular levels. The main aims of this study are: i) to infer the phylogenetic relationships of these two species, using molecular markers; ii) to compare their differentiation with the differentiation of all other species belonging to genus *Dolichopoda* previously analyzed (Allegrucci et al. 2005, 2009, 2011); iii) to revise the taxonomic arrangement of species and subspecies based on both morphological and molecular characters.

## Materials and methods

### Taxon sampling and laboratory procedures

**Figure 1.**
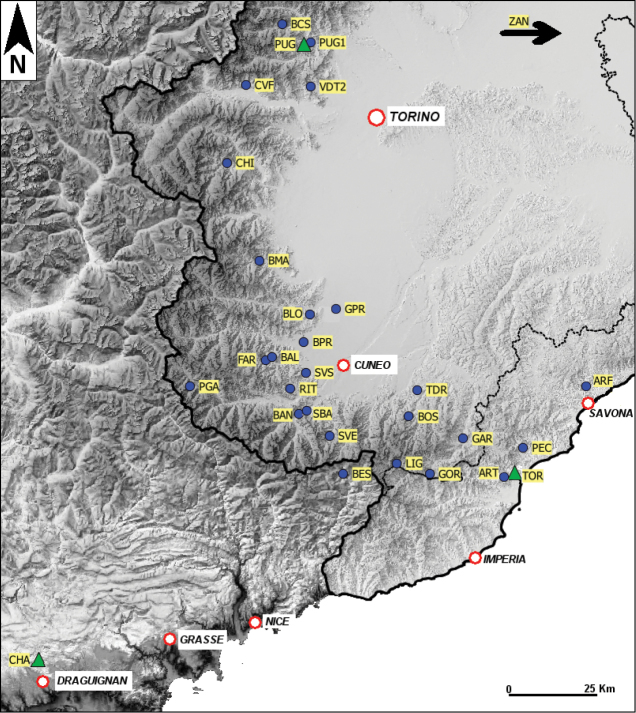
Sampling sites of *Dolichopoda* populations considered in this study (see also Table 1). Triangles indicate the type locality of the three considered taxa (CHA: *Dolichopoda azami*, TOR: *Dolichopoda ligustica ligustica*, PUG: *Dolichopoda ligustica septentrionalis*).

A total of 26 populations from Piedmont and Liguria (Maritime Alps, and western Liguria Apennines in northwest Italy), were analyzed in this study (Fig. 1, Table 1).

**Table 1.** North Western Italian *Dolichopoda* sampled in this study. All populations were analyzed at molecular level, while only the populations in which adults were present, were analyzed also at morphological level.

Taxon	Localities and sample size	Code	Morphology	GenBank Accession No.
*Dolichopoda azami*	Mine close to Chauves-souris Cave, Chateaudouble, Var, France, type locality, 525 m asl; Sampled individuals: 3	CHA	X	12S: KM086460; 16S: KM086485; COI: KM086535; 28S: KM086510
*Dolichopoda ligustica ligustica*	Taragnina Cave, Balestrino, SV, Liguria, close to Santa Lucia Inferiore Cave, 310 m asl; Sampled individuals: 5	ART		12S: KM086464; 16S: KM086489; COI: KM086539; 28S: KM086514
	Santa Lucia Inferiore Cave, Toirano, SV, Liguria, type locality, 194 m asl	TOR	X	
*Dolichopoda ligustica septentrionalis*	Borna Maggiore del Pugnetto Cave, Mezzenile, Lanzo Valley, TO, Piedmont, type locality, 820 m asl; Sampled individuals: 8	PUG	X	12S: KM086474; 16S: KM086499; COI: KM086549; 28S: KM086524
*Dolichopoda* sp.	Tana del Peccetto Cave, Magliolo, SV	PEC	X	
	Besta Cave, Vievola, Tenda, France, 910 m asl; Sampled individuals: 2	BES	X	12S: KM086459; 16S: KM086484; COI: KM086534; 28S: KM086509
	Falconiere Cave, Garessio,CN, Piedmont, 1000 m asl	GAR	X	
	Orso Cave, Ponte di Nava, Ormea, CN, Piedmont, 808 m asl; Sampled individuals: 3	GOR		12S: KM086472; 16S: KM086497; COI: KM086547; 28S: KM086522
	Viozene, Ormea, upper Tanaro Valley, CN, Piedmont (epigean), 1250 m asl; Sampled individuals: 2	LIG		12S: KM086470; 16S: KM086495; COI: KM086545; 28S: KM086520
	Vernante hypogeum, Vernante, Vermenagna Valley, CN, Piedmont, 800 m asl; Sampled individuals: 5	SVE	X	12S: KM086458; 16S: KM086483; COI: KM086533; 28S: KM086508
	Bossea Cave, Frabosa Soprana, Corsaglia Valley, CN, Piedmont, 836 m asl; Sampled individuals: 2	BOS	X	12S: KM086465; 16S: KM086490; COI: KM086540; 28S: KM086515
	Arma de Faie Cave, Albissola, Sansobbia Valley, SV, Liguria, 624 m asl	ARF	X	
	Bandito Cave, Valdieri, Gesso Valley, CN, Piedmont, 726 m asl; Sampled individuals: 2	BAN	X	12S: KM086471; 16S: KM086496; COI: KM086546; 28S: KM086521
	Bandito hypogeum, Roaschia, Gesso Valley, CN, Piedmont, 730 m asl; Sampled individuals: 7	SBA	X	12S: KM086463; 16S: KM086488; COI: KM086538; 28S: KM086513
	Dronera Cave, Vicoforte Mondovì, Ermetta Valley, CN, Piedmont, 525 m asl; Sampled individuals: 6	TDR	X	12S: KM086468; 16S: KM086493; COI: KM086543; 28S: KM086518
	Rittana Cave, Rittana, Stura di Demonte Valley, CN, Piedmont, 1000 m asl; Sampled individuals: 3	RIT	X	12S: KM086467; 16S: KM086492; COI: KM086542; 28S: KM086517
	Vallone Saben eastern hypogeum, Valdieri, Gesso Valley, CN, Piedmont, 750 m asl; Sampled individuals: 6	SVS	X	12S: KM086462; 16S: KM086487; COI: KM086537; 28S: KM086512
	Gaiola Cave, Gaiola, Stura di Demonte Valley, CN, Piedmont, 1020 m asl; Sampled individuals: 3	PGA	X	12S: KM086457; 16S: KM086482; COI: KM086532; 28S: KM086507
	Farout Cave, Pradleves, Grana Valley, CN, Piedmont, 1050 m asl; Sampled individuals: 3	FAR	X	12S: KM086461; 16S: KM086486; COI: KM086536; 28S: KM086511
	Balmarossa Cave, Pradleves, Grana Valley, CN, Piedmont, 1180 m asl; Sampled individuals: 2	BAL	X	12S: KM086452; 16S: KM086477; COI: KM086527; 28S: KM086502
	Buco del Partigiano Cave, Roccabruna, Maira Valley, CN, Piedmont, 1100 m asl; Sampled individuals: 7	BPR		12S: KM086455; 16S: KM086480; COI: KM086530; 28S: KM086505;
	Partigiani Cave, Rossana, Varaita Valley, CN, Piedmont, 615 m asl; Sampled individuals: 8	GPR	X	12S: KM086469; 16S: KM086494; COI: KM086544; 28S: KM086519
	Buco delle Locuste Cave, Pagliano, Varaita Valley, CN, Piedmont, 595 m asl; Sampled individuals: 2	BLO	X	12S: KM086466; 16S: KM086491; COI: KM086541; 28S: KM086516
	Buco del Maestro Cave, Paesana, Po Valley, CN, Piedmont, 750 m asl; Sampled individuals: 6	BMA		12S: KM086453; 16S: KM086478; COI: KM086528; 28S: KM086503;
	Chiabrano Cave, Perrano, Germanasca Valley, TO, Piedmont, 1080 m asl; Sampled individuals: 6	CHI	X	12S: KM086456; 16S: KM086481; COI: KM086531; 28S: KM086506
	Villar Focchiardo Cave, Villar Focchiardo, Susa Valley, TO, Piedmont, 460 m asl; Sampled individuals: 6	CVF		12S: KM086473; 16S: KM086498; COI: KM086548; 28S: KM086523
	Val della Torre Mine, Val della Torre, Casternone Valley, TO, Piedmont, 840 m asl; Sampled individuals: 5	VDT2		12S: KM086475; 16S: KM086500; COI: KM086550; 28S: KM086525
	Wolf Cave, Mezzenile, Lanzo Valley TO, Piedmont, 813 m asl; Sampled individuals: 4	PUG1	X	Same as PUG
	Borna del Servais Cave, Ala di Stura, Ala Valley, TO, Piedmont, 1440 m asl; Sampled individuals: 2	BCS		12S: KM086454; 16S: KM086479; COI: KM086529; 28S: KM086504;
	Lacca Selva Cave, Zandobbio, BG, Lombardy, 340 m asl; Sampled individuals: 2	ZAN	X	12S: KM086476; 16S: KM086501; COI: KM086551; 28S: KM086526

DNA was isolated from leg muscle of each individual, using a GenElute Mammalian Genomic DNA minipreparation Kit (Sigma-Aldrich, St. Louis, MO, USA) resuspended in 200 μl of sterile water and stored at -40 °C.

The entire Cytochrome Oxidase I gene (COI, total of 1500 bp), a 550-bp fragment of the 16S rRNA gene, a 450-bp fragment of the 12S rRNA gene and were amplified through the polymerase chain reaction (PCR) and sequenced from each individual. The large subunit of the nuclear ribosomal DNA (28S rRNA) was also included. The primers used were: LCO1490, HCO 2198 (Folmer et al. 1994), UEA1, UEA5 and UEA10 (Lunt et al. 1996) for the COI gene, 12Sai, 12Sbi (Kocher et al. 1989; Simon et al. 1994) for the 12S rRNA gene and 16Sar, 16Sbr (Simon et al. 1994) for the 16S gene. 28S rRNA was partially amplified and sequenced for a fragment of 580 base pairs, belonging to domains 3-5, using primers from Friedrich and Tautz (1997). Double-stranded amplifications were performed with a Perkin-Elmer-Cetus thermal cycler in 25 μl reaction volume containing genomic DNA (10–100 ng), 1.5 mM MgCl2, 0.2 mM of each dNTP, 0.1 μM primer, 1.5 units EuroTaq (Euroclone, UK) and the buffer supplied by the manufacturer. Optimal cycling parameters varied for each primer pair used. PCR products were purified using the ExoSAP digestion (Amersham Pharmacia Biotech), directly sequenced in both directions using the BigDye terminator ready-reaction kit, and resolved on ABI 3100 Genetic Analyzer (PE Applied Biosystems), following the manufacturer’s protocols. Sequence data were edited and compiled using CodonCode Aligner (version 3.7.1). All sequences were submitted to GenBank (Accession Numbers are reported in Table 1).

Each gene fragment (12S, 16S, COI and 28S) was considered separately for the alignment. Sequences of 16S, 12S and 28S were aligned using CLUSTAL_X 1.81 (Thompson et al. 1997) with opening gap = 10 and extending gap = 0.10. Cytochrome oxidase I nucleotide sequences were assembled, aligned, and translated with Codon-Code Aligner 3.7.1.

### Data analysis
Genetic differentiation and structure in the westernmost *Dolichopoda* species

Haplotype and nucleotide diversity were calculated using DNASP 5.10 (Rozas et al. 2003). Genetic distances between haplotypes and between groups of haplotypes were calculated using p-distance as implemented in MEGA 5.2 (Tamura et al. 2007). NETWORK 4.502 (Bandelt et al. 1999) was employed to calculate a median joining network representing the genealogical relationships among mtDNA haplotypes.

A Mantel test (Mantel 1967) with 5,000 simulations was used to test for an isolation-by-distance (IBD) signature (a positive correlation between geographic and genetic distances; Wright 1943; Slatkin 1993).

The hierarchical distribution of genetic variation was characterized using analysis of molecular variance (AMOVA). This method apportions genetic variation within and among groups, estimating F-statistics (Weir and Cockerham 1984; Excoffier et al. 1992; Weir 1996) that are analogous to Wright’s hierarchical fixation indices under the island model of gene flow (Wright 1951). Three-level AMOVA was conducted in ARLEQUIN 3.5.1.2 (Excoffier et al. 1992; Excoffier et al. 2005) using an *F*_ST_-like estimator. Samples were partitioned by geographic regions, populations within geographic regions, and populations. These tests included permutation of inferred haplotypes among groups (*F*_CT_); individual haplotypes among populations but within group (*F*_SC_); inferred haplotypes among populations (*F*_ST_).

### Species delimitation and phylogenetic analysis

Species delimitation analysis was carried out with two methods, a) the ”classical” DNA barcoding gap analysis (Hebert et al. 2003) and b) the Automatic Barcode Gap Discovery (ABGD, Puillandre et al. 2012). Both analyses were carried on the present samples and all *Dolichopoda* species previously analyzed (Table 2 and Allegrucci et al. 2005, 2011; Martinsen et al. 2009), considering only COI data set and only the common base pairs consisting of 964 bp.The ‘‘classical’’ DNA barcoding gap analysis was based on the pairwise genetic distances (p-distance). Distribution of genetic distance values was analyzed at different taxonomic levels.

**Table 2.** Species, populations, localities and accession numbers to GenBank for the *Dolichopoda* sequences included in this study and retrieved from GenBank for phylogenetic reconstruction of the western Mediterranean *Dolichopoda* species (Data from Allegrucci et al. 2005, 2009, 2011 and Martinsen et al. 2009).

Taxon	Localities	GenBank Accession No.
**Outgroup**		
*Euhadenoecus insolitus*	Indian Grave Point Cave (IND), De Kalb Co., TN, USA	12S: EF216947; 16S: AY793563; COI: AY793591; 28S: EF217005
*Hadenoecus cumberlandicus*	Bat Cave (BAT), Carter Cave State Park, Carter Co., KY, USA	12S: EF216948; 16S: AY793562; COI: AY793592; 28S: EF217004
*Troglophilus cavicola*	Covoli di Velo Cave (TRO), Veneto, Northern Italy	12S: EF216946; 16S: AY793564; COI: AY793624; 28S: EF217003
**Ingroup**		
*Dolichopoda bolivari*	Forat negre Cave, Serra del Llerida, Pyrenees, Spain	16S: AY507579-80, COI: AY507648-49
*Dolichopoda linderi*	Sirach Cave (SIR) Eastern Pyrenees, Western-South France	12S: JF826039; 16S: AY793567; COI: AY793598-99; 28S: JF826069
*Dolichopoda laetitiae*	Poscola Cave (PSC), Veneto, Northern-East Italy	12S: JF826054; 16S: AY793581; COI: AY793611 and 13; 28S: JF826076
	Piane Cave (GDP), Umbria, Central Italy	12S: JF826053; 16S: AY793582; COI: AY793610 and 12; 28S: JF826075
	Diavolo Cave (DIA), Tuscany, Central Italy	12S: JF826052; 16S: AY793580; COI: AY793614-15; 28S: JF826074
*Dolichopoda geniculata*	Valmarino Cave (VAL), Latium, Central-South Italy	12S: JF826055; 16S: AY793583; COI: AY793616-17; 28S: JF826077
	Fontanelle Cave (FON), Campania, Southern-West Italy	12S: JF826057; 16S: AY793584; COI: AY793594; 28S: JF826079
	Ischia cellars (ISC), Ischia Island, Campania, Southern-West Italy	12S: JF826058; 16S: AY793585; COI: AY793595; 28S: JF826080
	Roman Aqueduct (PNZ), Ponza Island, Latium, Southern-West Italy	16S: AY793586; COI: AY793596-97
*Dolichopoda capraensis*	San Michele Cave (CPR), Capri Island, Campania, Southern Italy	12S: JF826059; 16S: AY793587; COI: AY793606-7; 28S: JF826081
*Dolichopoda palpata*	Tremusa cave (TRE), Calabria, Southern Italy	12S: JF826060; 16S: AY793588; COI: AY793608-9; 28S: JF826082
*Dolichopoda baccettii*	Punta degli Stretti Cave (PST), Tuscany, Central-West Italy,	12S: JF826046; 16S: AY793571; COI: AY793639-40; 28S: JF826068
*Dolichopoda aegilion*	Campese Mine (CAM), Giglio Island, Tuscany, Central-West Italy	12S: JF826045; 16S: AY793570; COI: AY793600; 28S: JF826067
*Dolichopoda schiavazzii*	Pipistrelli Cave (ORS), Tuscany, Central-West Italy	12S: JF826044; 16S: AY793573; COI: AY793633; 28S: JF826066
	Marciana Cave (MRC), Elba Island, Tuscany, Central-West Italy	12S: JF826043; 16S: AY793572; COI: AY793635; 28S: JF826065
	Fichino Cave (FIC), Tuscany, Central-West Italy	12S: JF826042; 16S: AY793574; COI: AY793634-36; 28S: JF826064
*Dolichopoda bormansi*	Brando Cave (BRA), Corsica Island, France	12S: JF826047; 16S: AY793578; COI: AY793627 and 31-32; 28S: JF826069
	Sisco Cave (SIS), Corsica Island, France	12S: JF826048; 16S: AY793579; COI: AY793625-26 and 28; 28S: JF826070
*Dolichopoda cyrnensis*	Valletto Cave (VLT), Corsica Island, France	12S: JF826050; 16S: AY793577; COI: AY793620-21; 28S: JF826072
	Sabara Cave (SAB), Corsica Island, France	12S: JF826049; 16S: AY793576; COI: AY793618-19; 28S: JF826071
*Dolichopoda muceddai*	Limbara Mount, Sardinia Island, Italy	12S: JF826051; 16S: AY793575; COI: AY793629-30; 28S: JF826073

The ABGD method (Puillandre et al. 2012) automatically finds the distance at which a barcode gap occurs and sorts the sequences into putative species based on this distance. The method statistically infers the barcode gap from the data and partitions the data accordingly. Populations belonging to the same species therefore should be grouped in the same partition. This procedure is then recursively applied to the previously obtained groups of sequences. COI alignments were uploaded at http://wwwabi.snv.jussieu.fr/public/abgd/abgdweb.html and ABGD was run with the default settings (*P* min = 0.001, *P* max = 0.1, Steps = 10, X (relative gap width) = 1.5, Nb bins = 20) and using p-distance.

A phylogenetic analysis was carried out considering the four sequenced genes, by constructing a concatenated matrix, partitioned by genes. Phylogenetic analysis was performed only on the western Mediterranean *Dolichopoda* species, using Bayesian inferences as implemented by the software MrBayes 3.1b4 (Huelsenbeck and Ronquist 2001). Mrmodel test (Nylander 2004) was used to perform hierarchical likelihood ratio test and calculate approximate Akaike Information Criterion (AIC) values of the nucleotide substitution models for each gene fragment.

At least two simultaneous searches were conducted comprising four Markov chains started from a randomly chosen tree and run for 1,000,000 generations, with sampling every 100 generations. The following descriptors were assumed to indicate convergence on a common phylogenetic topology by separate Bayesian searches: similarity in log likelihood scores at stationarity, similarity in consensus tree topologies and PP values for supported nodes, and a final average standard deviation of split frequencies (ASDSF) for simultaneous searches approaching zero. The first 1,000 trees were discarded as burn-in and posterior probabilities (PP) were calculated from post burn-in trees.

### Morphological analysis

The most important morphological characters used in the systematics of the genus *Dolichopoda* are the shape of the epiphallus and of the male tergum 10. In this study, the investigation of these characters was integrated by the analysis of other morphological features as male sub-genital fig, female sub-genital fig and shape of ovipositor.

Overall, we have checked 22 populations from the northwestern Italy for a total of 64 adult individuals. All these populations are the same analyzed for the genetic variation (Table 1). Further comparisons were made using three more samples (PEC, ARF, GAR) coming from the collection of Museum of Natural History of Genoa G. Doria. The specimens were studied using a stereomicroscope Leica MZ 12.5. All the measures of the morphological parameters are in mm. Photos were taken with a digital camera, Nikon Coolpix P300. Pictures were processed using a digitiser board WACOM CTH 461 and Adobe Photoshop CS3 Extended Version 10.0.

Table 3 shows the measures of twelve morphological characters in 6 populations where a sufficient number of adults were available. In particular, we considered the total body length, the pronotum length, and the length of tarsum, femur and tibia of the fore, middle and hind legs in both males and females and the ovipositor length in females. Measurements were taken using a digital caliper (accuracy 0.01 mm). Morphometric variables were analyzed by using the non-parametric ANOVA Kruskal-Wallis test. A multiple regression analysis was carried out comparing morphometric variables with the altitude of the caves.

**Table 3.** Measures (min and max in mm) of 12 morphological characters in six samples of northwestern Italian *Dolichopoda* taxa.

	CHA		BES		CHI		FAR		TOR		PEC	
	♂ n=4	♀ n=3	♂ n=4	♀ n=2	♂ n=4	♀ n=4	♂ n=4	♀ n=5	♂ n=3	♀ n=4	♂ n=2	♀ n=1
Body	16.0–16.5	17.0–18.0	14.0–16.0	14.0–15.0	15.0–16.0	15.0–17.0	14.0–15.5	15.0–18.0	17.0–21.0	19.0–21.0	16.0–17.0	16.0
Pronotum	4.0–5.0	4.0	3.5–4.0	3.5–4.0	3.8–4.0	4.0	4.0–4.5	4.0–4.5	4.0	4.0–4.5	4.0	4.0
Fore tibia	15.0–17.0	14.5–15.0	14.5–17.0	13.0–14.0	14.5–15.5	13.0–14.0	14.0–16.0	13.5–15	17.0–18.5	15.0–17.0	14.0–15.5	14.5
Fore femur	15.5–16.0	14.0	14.0–15.5	12.0–13.0	14.0–15.0	12.0–13.0	13.0–15.0	12.0–14.0	14.5–17.0	14.0–16.0	14.0–15.0	14.0
Fore tarsum	7.0–7.5	6.0–7.0	6.0–7.0	5.0	6.0–7.0	5.0–6.0	5.0–6.5	5.0–6.0	6.0–7.5	6.0–7.0	5.5–6.5	6.0
Medium tibia	17.0	14.5–16.0	15.0–16.5	13.0–14.0	15.0–16.0	13.0–14.0	13.0–16.0	13.5–15.0	17.0–19.0	16.0–17.0	14.0–16.0	15.0
Medium femur	16.0–16.5	14.0	14.5–15.5	12.0	14.0–15.0	12.0–13.0	13.0–15.0	12.5–14.0	15.0–17.0	14.0–16.0	14.0–15.0	13.0
Medium tarsum	6.0	5.0	5.0–6.0	5.0	5.0–5.5	4.5–5.0	5.0–6.0	4.5–5.0	6.0–6.5	5.0–6.0	5.5–6.0	5.0
Hind tibia	30.0–32.0	30.0–34.0	25.0–29.0	25.0–27.0	23.5–27.0	24.0–25.0	24.5–27.5	25.0–27.5	26.5–34	30.0–32.0	27.0–29.0	28.0
Hind femur	25.5–26.5	25.0–26.5	22.5–24.5	22.0–23.0	21.0–24.0	22.0–22.5	22.0–24.0	22.0–23.5	24.0–27.0	23.5–27.0	22.0–24.0	24.0
Hind tarsum	6.0–7.0	6.0	5.5–6.0	5.0–6.0	4.0–6.0	4.0–5.5	5.5–6.0	5.0	6.0–7.0	5.5–6.0	5.5–6.5	5.5
Ovipositor		13.0–14.0		11.0		12.0–13.0		10.0–13.0		11.0–13.0		12.0

## Results

### Genetic differentiation and structure in the westernmost *Dolichopoda* species

A total of 2450 mitochondrial base pairs corresponding to the entire *COI* gene, to 450 bp of 12S and 550 bp of 16S were successfully sequenced and aligned in 104 specimens of *Dolichopoda* populations from northwestern Italy. 28S rRNA, consisting of 500 bp was also sequenced in all considered samples. Ribosomal genes were identical in all assayed samples. Therefore, the analysis was limited to the COI mitochondrial gene.

The 104 individual COI sequences comprised a total of 15 haplotypes determined by 13 segregating sites. Global haplotype diversity, *h*, was 0.875 and nucleotide diversity, *p*, was 0.00231.

**Figure 2.**
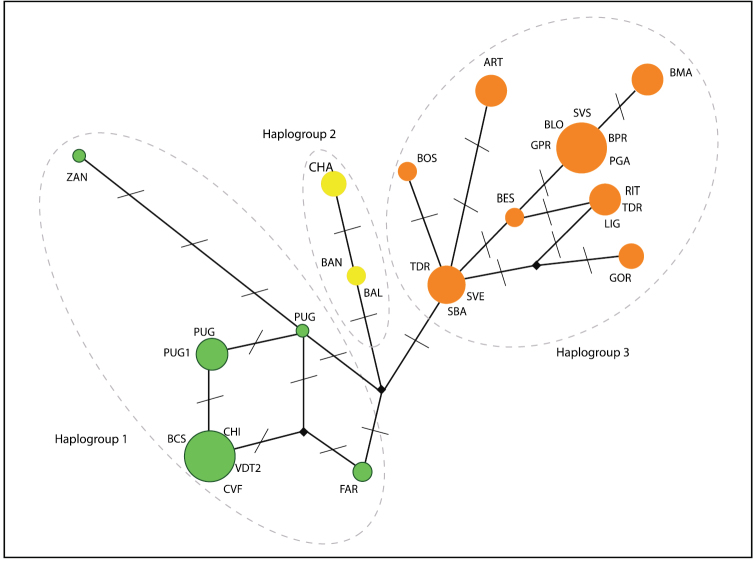
Median-joining network analysis in the populations of *Dolichopoda* considered in this study: circled areas are proportional to the number of individuals sharing the same haplotype; bars, along connections, indicate the number of nucleotide substitutions. Individuals from the same population can show different haplotypes. Based on number of nucleotide substitutions, three haplogroups can be distinguished, each indicated with different color.

*Dolichopoda* haplotypes were organized in three main haplogroups in which haplotypes are ordered geographically (Fig. 2). In particular, haplogroup 1 included samples from the northernmost area, haplogroup 2 samples from the westernmost area and haplogroup 3 consisted of samples from the south-eastern area (Fig. 2).

**Figure 3.**
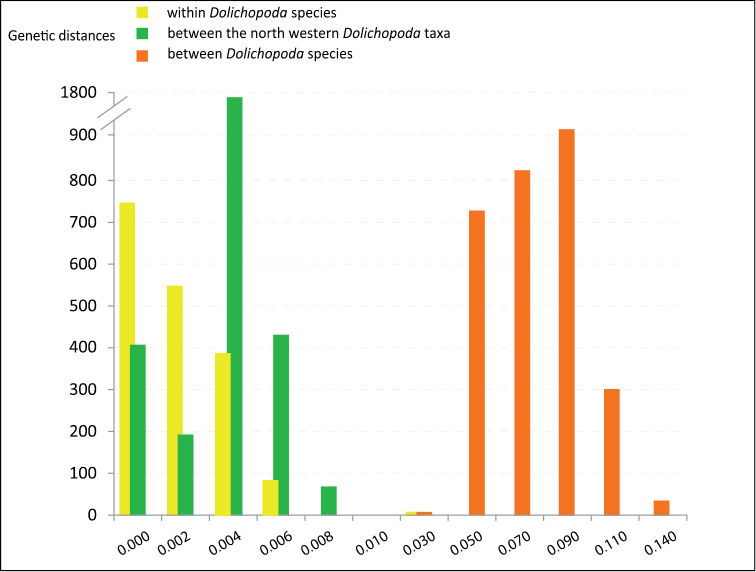
Distribution of genetic distance values (p-distances) at different taxonomic levels. Pairwise comparisons at the intra- and inter-specific level in all *Dolichopoda* species are reported.

To investigate this further we carried out a genetic distance analysis using COI gene as a barcode and p-distance between all studied *Dolichopoda* species (Allegrucci et al. 2005, 2009, 2011; Martinsen et al. 2009). Genetic distance values at intra- and interspecific levels are compared in Figure 3. In particular, Figure 3 shows the genetic distance values found in intra- and interspecific comparisons of all studied *Dolichopoda* species compared to the interspecific values found in comparisons between the northwestern Italian populations. Intraspecific values between all *Dolichopoda* species ranged from 0 to 0.023, with a mean of 0.001(± 0.0019, standard deviation), while interspecific values ranged from 0.016 to 0.134, with a mean of 0.134 (± 0.020, standard deviation). Pairwise comparisons between the northwestern Italian populations ranged from 0 to 0.007 with a mean of 0.003 (± 0.0015, standard deviation).

ABGD analysis proposed several partitions that varied according to the different a priori thresholds. Apart from the two extreme a priori threshold values (*P* = 0.001 and *P* = 0.035), for which aberrant number of species hypotheses were obtained (almost every haplotype was considered as a different species hypothesis for *P* = 0.001 and, conversely, all the haplotypes were combined in a single species hypothesis for *P* = 0.035), all the tested a priori thresholds lead to the same splitting. The westernmost *Dolichopoda* taxa were grouped together, while the other groups corresponded to the nominal species.

Analysis of molecular variance (AMOVA; Excoffier et al. 1992) used to measure the proportion of genetic variation among subdivided populations, suggested a strong structure within the populations (*F*_ST_ = 0.979, *P*=0). Genetic variation among different geographic groups and among populations within each group was 52.16 % and 45.8 %, respectively, with *F*_CT_ = 0.522 (*P* = 0) and *F*_SC_ = 0.957 (*P* = 0). Mantel test suggested a clear isolation by distance across the sampled region (R^2^ = 0.623, *P* ≤ 0.002).

### Phylogenetic analysis

**Figure 4.**
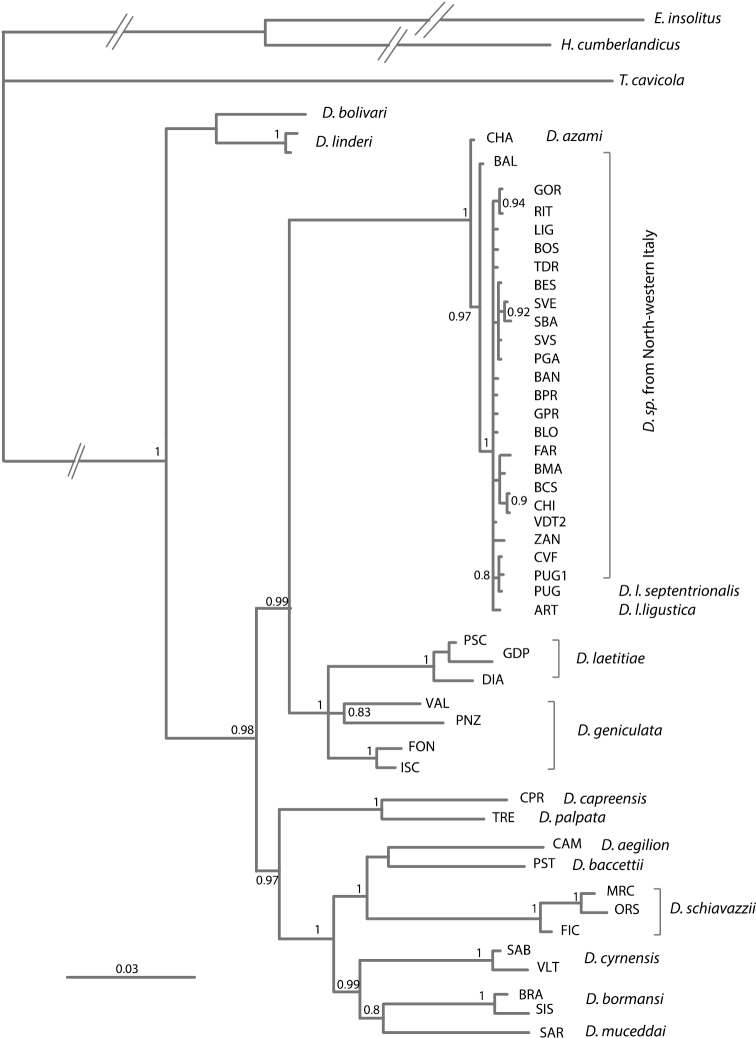
Relationships among Italian species of *Dolichopoda* inferred from Bayesian analysis based on three mitochondrial and one nuclear genes. Values above branches indicate posterior probabilities derived from Bayesian analysis. Scale bars: 0.03 substitutions per site. Only posterior probability (PP) values ≥ 0.80 are shown.

MRMODELTEST (Nylander 2004) indicated GTR + I + G (Lanave et al. 1984; Gu et al. 1995) and K80 + I (Kimura 1980) as the best models of DNA substitution for the mitochondrial genes and the nuclear one, respectively. The phylogeny in Figure 4 supports the major phylogenetic relationships previously demonstrated (Allegrucci et al. 2005, 2011; Martinsen et al. 2009). The Italian *Dolichopoda* species are separated in two main clusters, as expected. The first group included the continental species from Liguria to southern Italy (*Dolichopoda ligustica*, *Dolichopoda ligustica septentrionalis*, *Dolichopoda azami*, *Dolichopoda laetitiae* and *Dolichopoda geniculata*). Populations from *Dolichopoda ligustica*, *Dolichopoda ligustica septentrionalis*, and *Dolichopoda azami* constituted a homogeneous clade well differentiated from *Dolichopoda laetitiae* and *Dolichopoda geniculata*. The second group was represented by the southernmost species from Tuscany, to Corsica, Campania and Calabria (i.e., *Dolichopoda aegilion*, *Dolichopoda baccettii*, *Dolichopoda schiavazzii*, *Dolichopoda cyrnensis*, *Dolichopoda bormansi* and *Dolichopoda muceddai* and *Dolichopoda palpata* with its sister taxon *Dolichopoda capraensis*). Linked to these clusters are *Dolichopoda linderi* and *Dolichopoda bolivari*.

### Morphological analysis

**Figures 5–13.**
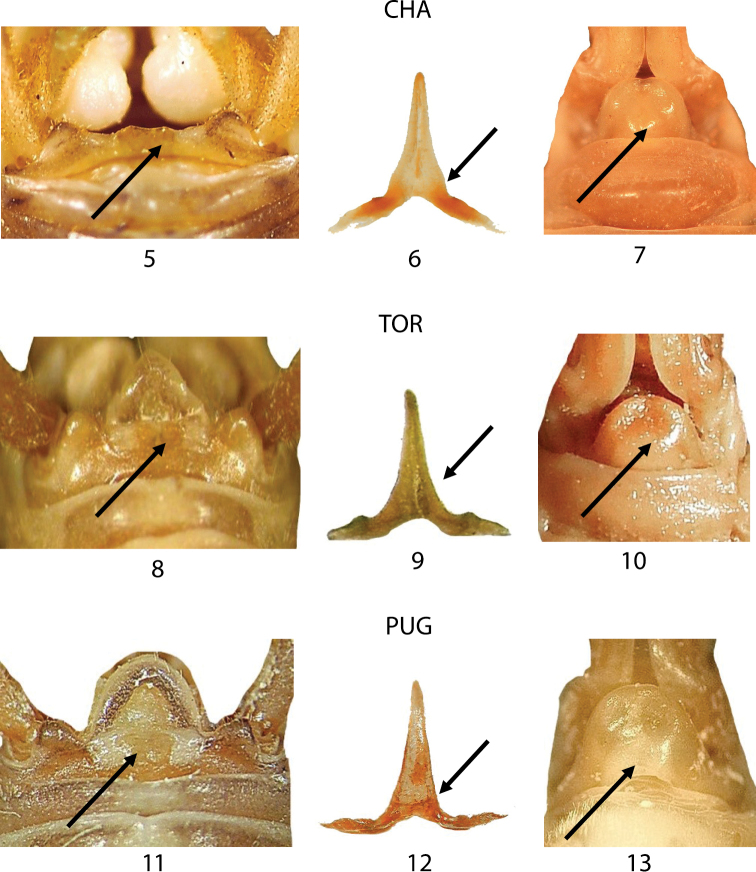
Morphological characters of *Dolichopoda azami* (CHA type locality), *Dolichopoda ligustica ligustica* (TOR type locality) and *Dolichopoda ligustica septentrionalis* (PUG type locality). Left: dorsal view of tergum 10; centre: epiphallus of the male; right: particular of the female sub-genital fig. Arrows show diagnostic characters.

In Figures 5–13 morphological comparisons among the three samples from the typical locality of *Dolichopoda azami* (Chateaudouble, CHA), *Dolichopoda ligustica* (Toirano, TOR) and *Dolichopoda ligustica septentrionalis* (Pugnetto, PUG) are reported. In these figures, the three main characters are compared: male tergum 10, epiphallus and female sub-genital fig. The other two considered morphological features, male sub-genital fig and ovipositor, are not reported because showed a very uniform pattern. As already evidenced in the taxonomic revision of the genus *Dolichopoda* (Baccetti and Capra 1959), it is possible to distinguish the species *Dolichopoda azami* through the male tergum 10 that shows two rounded lobes separated by a large concavity with two little acuminate tips (Fig. 5) and median process of the epiphallus enlarged at the basis (Fig. 6). In the female, the sub-genital fig is completely rounded with a narrow apical incision (Fig. 7). On the contrary, *Dolichopoda ligustica* shows a male tergum 10 with little oval lobes separated by almost straight margin (Fig. 8), and median process of epiphallus pyramidal with lateral margin almost straight (Fig. 9). The female sub-genital fig is very similar to that of *Dolichopoda azami* (Fig. 10) but the median incision appears less pronounced. Finally, the subspecies *Dolichopoda ligustica septentrionalis* is distinguishable only for the more slender median process of the epiphallus (Fig. 12). Based on these observations, it is evident the great similarity of the three taxa here considered and consequently the strong difficulty in their morphological distinction.

In Figures 14–22, we compared three samples from western Liguria (Table 1). The three considered characters appear uniform resembling all to the morphology of TOR (Figs 8–10) the typical *Dolichopoda ligustica*. On the contrary, the analysis of a series of samples from northern and southern Piedmont (Figs 23–37) highlights a more complex situation with the contemporary presence of characters typical of one or the other species in the same individual. In particular, even if all the specimens analyzed show the tergum 10 very similar to that of *Dolichopoda azami* (cfr Fig. 5 with Figs 23, 26, 29, 32, 35) the same specimens often show the shape of epiphallus and of the female sub-genital fig more similar to that of *Dolichopoda ligustica*.

**Figures 14–22.**
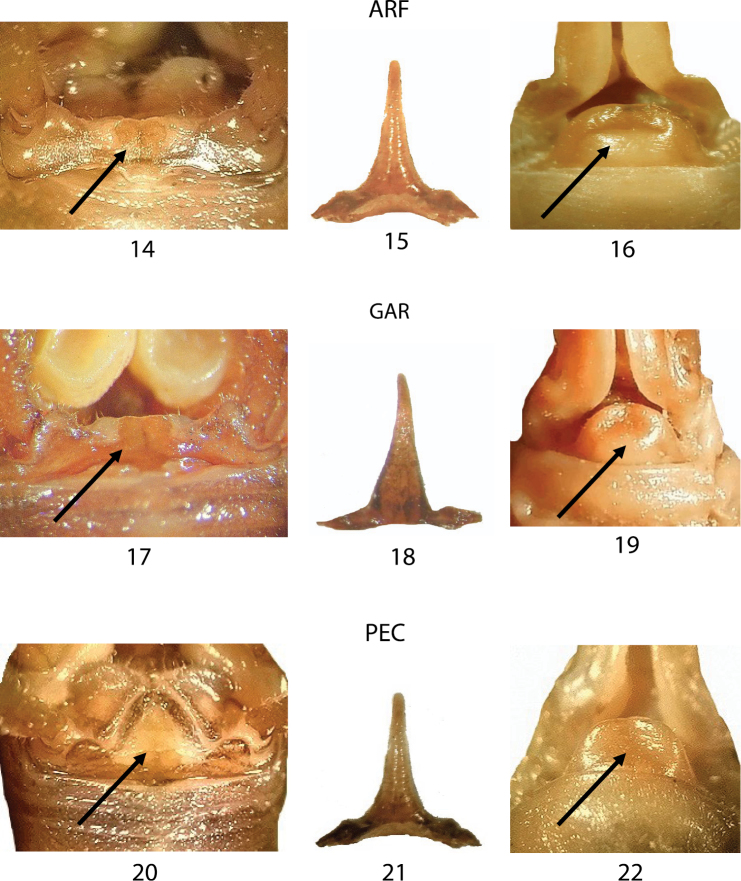
Morphological characters of the *Dolichopoda* from Liguria: ARF, GAR and PEC. Left: dorsal view of tergum 10; centre: epiphallus of the male; right: particular of the female sub-genital fig. Arrows show diagnostic characters.

**Figures 23–37.**
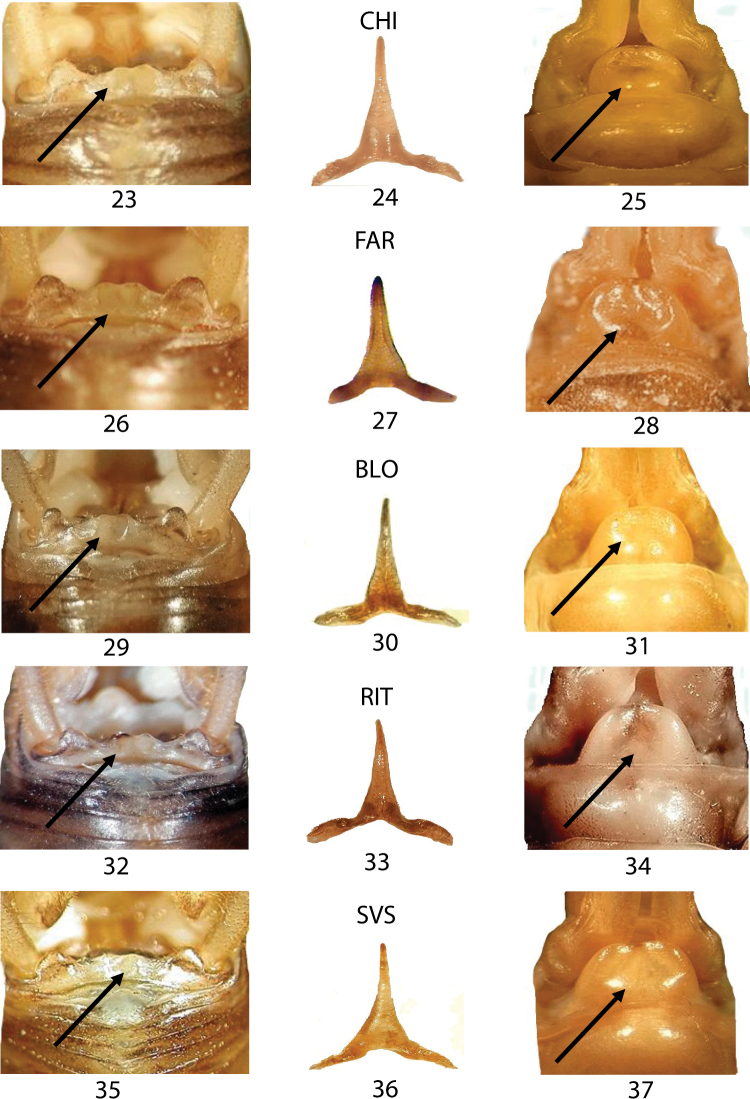
Morphological characters of the *Dolichopoda* from Piedmont: CHI, FAR, BLO, RIT and SVS. Left: dorsal view of tergum 10; centre: epiphallus of the male; right: detail of the female sub-genital fig. Arrows show diagnostic characters.

In Table 3, the measures of 12 morphological characters, recorded on 6 populations, where a sufficient number of adults were available, are reported. Mann-Whitney test carried out between males and females morphometric traits revealed significant differences (*P* < 0.05), being the females smaller than males. ANOVA analysis highlighted significant differences between some morphometric measures in specimens coming from caves situated at different altitude both in males and in females. Multiple regression analysis revealed a significant correlation between the morphometric measures and the altitude of the cave (R^2^ = 0.661, Wilk’s lbd = 0.0045; *P* << 0.01). In particular, samples coming from caves at low altitudes resulted bigger than those from caves at higher altitudes.

## Discussion

The Northwestern Italian *Dolichopoda* species group comprises a uniform assemblage of populations, both genetically and morphologically. From genetically point of view, the median joining network analysis (Fig. 2) evidenced three main haplogroups geographically distributed, but the resulting scenario is rather homogeneous with a maximum number of mutation events equal to 8. Genetic distance values based on COI gene revealed a very little differentiation between all studied samples. All pairwise comparisons fall in the range of intraspecific comparisons observed in all the other *Dolichopoda* species, with a mean of 0.3% (Fig. 3). These results suggest a level of differentiation usually found between populations belonging to the same species rather than to different species. ABGD analysis confirms these results, grouping together all the northwestern *Dolichopoda* taxa.

However, despite the low variability, these populations show a significant level of genetic structure. Indeed, AMOVA analysis evidenced significant partitioning of variation within and among populations and among regions and a low level of gene flow between the different regions. Isolation by distance was also detected by Mantel test, suggesting that dispersal rates in these *Dolichopoda* populations are very low and limited to the geographically close ones. This result is not surprising for cave dwelling organisms because it reflects the geographic isolation of the different caves and / or groups of caves to which *Dolichopoda* populations are confined (Sbordoni et al. 2012). The Maritime Alps region is characterized by a combination of Mediterranean, Apennine and Alpine flora and this diversity in habitat geography could strongly influence the different degree of isolation of the *Dolichopoda* populations. While the presence of beech and oak forests would account for continuing gene flow among *Dolichopoda* species, the occurrence of Alpine forests and to some extent of the Mediterranean “macchia” could prevent the gene exchange (Allegrucci et al. 1997; Sbordoni et al. 1985, 2012).

The phylogeny illustrated in Figure 4, based on three mitochondrial (COI, 12S and 16S) and one nuclear (28S) genes, supports the major phylogenetic relationships previously demonstrated (Allegrucci et al. 2005, 2011) highlighting that the northwestern Italian populations represent a single, uniform, well supported cluster. With respect to the other Italian species, they are genetically very similar, showing very short branch lengths and forming a single cluster in which the relationships between the different populations are not clearly resolved. Based on previous studies carried out on the 90% of the species belonging to genus *Dolichopoda* (Allegrucci et al. 2005, 2011), we know that divergence time between these populations and their sister taxon, represented by the cluster *Dolichopoda laetitiae*–*Dolichopoda geniculata*, is dated back to about 2 Million years ago, during the late Pliocene. In that time, the western Mediterranean lineages probably arose from a single colonization event from the North of Italy. An ancestral group spread in two directions, colonizing on one hand Piedmont, Liguria and France and, on the other side, the entire inland of Italian Peninsula.

The morphological analysis shows high degree of similarity between all analyzed samples, suggesting that the morphological traits commonly used in the taxonomy of the genus *Dolichopoda* (tergum 10, epiphallus shape in the male and sub-genital fig in the female), are not able to discriminate clearly specimens belonging to *Dolichopoda azami*, *Dolichopoda ligustica* and *Dolichopoda ligustica septentrionalis*. In particular, although very few morphological differences can be observed between specimens from the typical localities of the three taxa (CHA, typ. loc. of *Dolichopoda azami*; TOR, typ. loc. of *Dolichopoda ligustica* and PUG, typ. loc. of *Dolichopoda ligustica septentrionalis*), the most of the studied samples show some variability in the diagnostic characters occurring more or less randomly. Often we observed in the same specimen, the contemporary presence of characters typical of one or the other taxon.

Morphometric analysis suggested significant differences both between males and females and between samples from caves situated at different altitudes. Caves situated at high altitude are presumably subjected at lower temperatures and therefore the bioclimatic factors may be the major determinants of the morphometric patterns observed. Morphological traits might be subjected to substantial non-genetic variation and/or might show local short-term adaptations to peculiar environmental conditions. These differences were already been observed in populations of *Dolichopoda laetitiae* and *Dolichopoda geniculata* in the central southern Italy (Allegrucci et al. 1987; Cesaroni et al. 1997). In those studies, we demonstrated that variations in morphometric measures (and in particular in leg elongation) is almost entirely under the control of an environmental gradient, synthetically described by the cave temperature.

In conclusion, the scenario, depicted by both molecular markers and morphological traits, suggests that the northwestern Italian *Dolichopoda* constitute a single species with populations characterized from high similarity between each other and, due to the priority of description, they should be assigned to *Dolichopoda azami* Saulcy, 1893 which is the older available name at the species rank. However, in order to portrait the geographical partitioning and the related genetic differences we suggest to preserve the names *ligustica* Baccetti & Capra, 1959 and *septentrionalis* Baccetti & Capra, 1959 to indicate groups of populations corresponding to the different COI haplogroups as outlined in the network analysis (Fig. 2).

Therefore, the new taxonomic arrangement here proposed is:

***Dolichopoda azami azami* Saulcy, 1893**

**Type locality.** Mine close to Chauves-souris Cave, Chateaudouble, Var, France, 525 m asl;

***Dolichopoda azami ligustica* Baccetti & Capra, 1959, stat. n.**

**Type locality.** Santa Lucia Inferiore Cave, Toirano, SV, Liguria, 194 m asl;

***Dolichopoda azami septentrionalis* Baccetti & Capra, 1959, stat. n.**

**Type locality.** Borna Maggiore del Pugnetto Cave, Mezzenile, Lanzo Valley, TO, Piedmont, 820 m asl.
